# Development and regulatory approval of Kal91.3: combining advanced conventional breeding with genetic engineering to create a cold sweetening‑resistant chip‑processing potato

**DOI:** 10.1080/21645698.2026.2707844

**Published:** 2026-07-28

**Authors:** Kelly A. Zarka, Nanye Long, Joseph Coombs, David S. Douches

**Affiliations:** a Department of Plant, Soil and Microbial Sciences, Michigan State University, East Lansing, MI, USA; b Institute for Cyber Enabled Research, Michigan State University, East Lansing, MI, USA; c Bioinformatics Core, Michigan State University, East Lansing, MI, USA

**Keywords:** Gene silencing, potato common scab, potato transformation, regulatory review, vacuolar acid invertase

## Abstract

Globally, potato (*Solanum tuberosum*) is an important food crop. Due to an expanding potato‑chip processing market, it is increasingly essential that high-quality raw tubers are maintained and available year-round. Cold storage at temperatures below 10°C greatly increases the duration of potato tuber storage, however, potatoes are susceptible to cold-induced sweetening (CIS). At these temperatures, potato starch stored in the tuber converts to reducing sugars, leading to a dark, bitter-tasting potato chip. Kalkaska is a potato variety with high yield and a highly desirable scab resistance; however, it is unreliable in managing reducing sugars in commercial storage. In this study, we report a comprehensive characterization and analysis of a genetically engineered potato line Kal91.3, utilizing *VInv* RNAi suppression to correct the reducing sugar management in Kalkaska. A full molecular characterization analysis was completed on Kal91.3, which demonstrated that there are two independent and stable T-DNA insertions. RNA analysis reported suppression of the *VInv* transcripts, which was further shown during a prolonged tuber storage period. Kalkaska and Kal91.3 tubers were stored at 4°C for up to 8 months. Kal91.3 produced chips light in color, and the Kalkaska control produced dark chips typical of the CIS darkening response. Kal91.3 is the first genetically engineered field‑crop product developed by a non-industry organization and to have received regulatory clearance in the United States. Kal91.3 is expected to provide significant benefits to growers, processors, and consumers by producing high-quality potato chips from long-term, lower-temperature storage, improving supply‑chain stability, and waste reduction.

## 1. Introduction

Potato (*Solanum tuberosum* L.) is the third most important crop worldwide, behind rice and wheat.^1^ Potatoes are purchased as fresh market produce and in processed forms, including frozen, chips, dehydrated, and canned. By 2031, the global potato chip industry market is expected to increase to over $80 billion USD.^2^ Year-round supplies of potatoes, ready for chip processing, are needed even though many of the growing regions around the world are only able to have one potato growing season each year. To efficiently meet market demand, the industry must incorporate effective ways to mitigate supply constraints.

Potato storage, which requires specific temperature, humidity, and ventilation control, is a major industry constraint.^3^ Large potato storage facilities try to reduce losses by storing potatoes at low temperatures below 10°C (55°F) to prevent sprouting and delay bacterial and fungal growth. However, the cold temperatures needed for better storage also promote the accumulation of reducing sugars. Cold temperatures below 10°C (55°F) trigger enzymes within the potato tuber to convert starch into reducing sugars in a process called cold-induced sweetening (CIS).^4^ When potatoes with CIS are sent for chip processing, the elevated sugars react with free‑radical α-amino acids via the Maillard reaction during the frying process.^5^ The higher the sugar content, the darker the chip color, and this makes chips bitter-tasting and unmarketable.

Due to the high economic value of quality light-colored potato chips, research into the mechanism and pathways involved in CIS has been well documented^5,6^ and.^7^ Although complex pathways are involved, CIS is driven, for the most part, by the cold-responsive activation of vacuolar invertase. This enzyme catalyzes the breakdown of sucrose into glucose and fructose. Traditional potato‑breeding research has focused on selecting cultivars that have low reducing‑sugar content, and to some extent, the results have reduced color darkening during frying.^8^ Further research determined that RNAi silencing method could be used to prevent CIS in cultivated potato.^9^ Similarly, the same team of scientists at the University of Wisconsin Madison, Wu et al.^10^ published on using RNAi invertase silencing on four potato cultivars popular in the United States. They selected lines that had a reduction of *Vinv* transcripts of >90%. The results showed that reducing sugars were minimally accumulated during cold storage, and that potato chips were light colored compared to the controls after cold storage. Field results showed no changes in growth and yield in association with *VInv* suppression.

RNAi silencing has been used to lower invertase levels in commercial potato varieties. Zhu et al.^11^ showed that browning and acrylamide formation during French fry processing were reduced in the potato cv. Russet Burbank. The cultivar Katahdin was used by^12^ to demonstrate that *VInv* silencing can successfully suppress CIS in potatoes stored at 3°C for up to 8 months, and Hameed et al.^13^ showed that CIS can be suppressed in cv. Desiree. Although these commercial varieties containing CIS resistance were developed, none of them were deregulated or commercially released. However, J.R. Simplot Company’s Simplot Plant Science’s (Boise, Idaho USA) division developed the “Innate” potato line, and not only did they include RNAi silencing for low‑temperature storage and low acrylamide potential, but later Innate versions included other trait enhancements such as late blight resistance, reduced black spot and browning, and reduced asparagine. Simplot Plant Sciences focused on well‑known host varieties like Russet Burbank, Ranger Russet, Atlantic, and Snowden.^14^ Innate potato lines were deregulated through the United States Department of Agriculture/APHIS (USDA). These submissions are publicly available.^15^ The Food and Drug Administration (FDA) has also reviewed Simplot Plant Science’s downregulation of vacuolar invertase (*VInv*) using RNAi in Biotechnology Notification Files (BNF) 146, 153, 174, and 197.^16^

Regulatory approvals of genetically modified (GM) crops are completed by national authorities that require comprehensive evaluations to confirm the safety of the genetically modified crop under review. Since the first GM crop approvals in the late 1990s, the data and methodologies used in the assessments have become increasingly standardized. Today, these evaluations follow internationally recognized guidance, most notably the *Codex Alimentarius Guideline for the Conduct of Food Safety Assessment of Foods Derived from Recombinant-DNA Plants*.^17^

In this study, we incorporated Bhaskar et al.^9^ RNAi invertase silencing technology into Kalkaska, one of our advanced conventionally bred scab‑resistant potato varieties. Kalkaska is a high-yielding, chip-processing variety with valuable traits, including common scab resistance and tolerance to black‑spot bruises.^18^ Kalkaska produces medium-set, uniform, round white chip-processing tubers with a low level of internal defects. The yields are high across many environments.

Here, we share the development of the transformed Kalkaska event designated as Kal91.3 and the collection of studies that were completed and submitted to the United States regulatory agencies. The data include molecular characterization, trait analysis, agronomic analysis, and compositional analysis. The information was collected and submitted as part of a Regulatory Submission Review (RSR) through the USDA following their guidelines.^19^ The USDA implemented the RSR, in Oct. 2021, under a new Sustainable, Ecological, Consistent, Uniform, Responsible, Efficient (SECURE) system for the evaluation of new genetically modified plants, replacing 7 C.F.R. Part 340. Exemptions for previously reviewed genes and gene combinations were possible. However, even though the RNAi invertase technology was previously reviewed and approved by USDA APHIS with Simplot Plant Science’s Innate potato products, our submission for Kal91.3 used a different promoter and a different selectable marker. Therefore, it was not eligible for an exemption, and a submission requiring an RSR was needed. Additionally, our scientific and regulatory assessment of Kal91.3 was submitted to the FDA as a Biotechnology Notification File (BNF000205) through the FDA’s Biotechnology Final Consultation Food Safety Evaluation for New Plant Variety Consultations guidelines.^20^

## 2. Materials and Methods

### 2.1. Vector Construction

**Figure 1.**
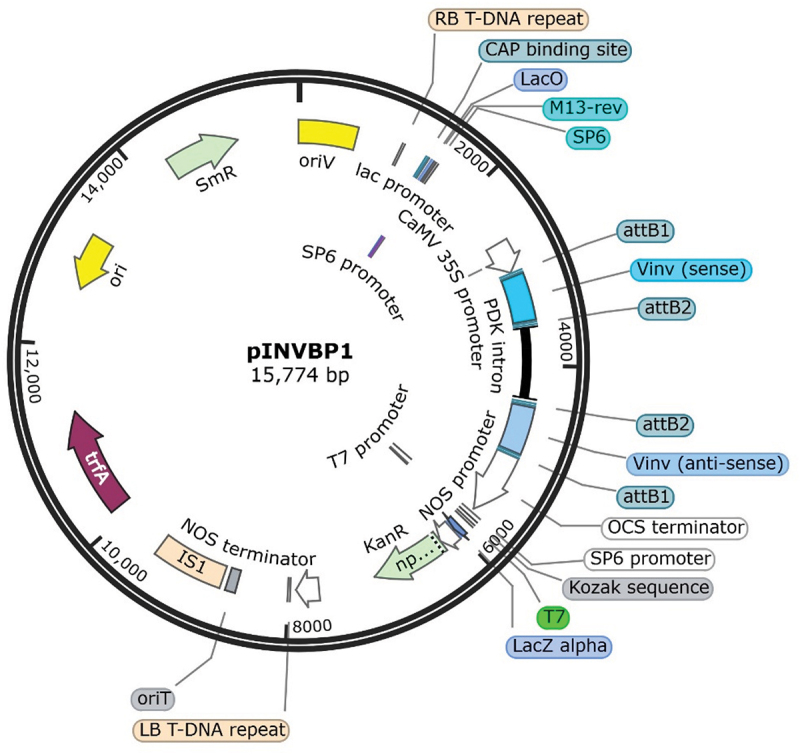
Plasmid map of pINVBP1.

The plasmid construct pINVBP1 was used for an *Agrobacterium*-meditated potato transformation, which contains 508 bp of potato *VInv* cDNA and uses the plasmid pHELLSGATE 8 as its base.^9^ Information on all of the genetic elements in plasmid pINVBP1 can be found in the Supplementary Table S1. The pINVBP1 vacuolar invertase silencing cassette utilizes the commonly used constitutive 35S CaMV promoter. A plasmid map was created using SnapGene software (http://www.snapgene.com) (Figure 1). The plasmid and T-DNA sequences are provided in the Dryad repository, accession number doi: http://doi.org/10.5061/dryad.rjdfn2zsm

Schematic representation of the plasmid construct pINVBP1 used for the transformation of Kalkaska. Created using SnapGene software (http://www.snapgene.com).

### 2.2. Transformation

Kalkaska was used for transformations with the pINVBP1 plasmid. Potato plant events were produced using *Agrobacterium-mediated* transformation at MSU. The *Agrobacterium tumefaciens* strain GV3101 carrying pINVBP1 was used to transform potato internode explants following the method described by Douches et al.^21^ Transformed internode explants were regenerated on medium containing 50 mg/L kanamycin to select for lines containing a T-DNA insert.

### 2.3. Selection of Kal91.3 Event

Plantlets that visually grew well and rooted in the selective media were transferred to soil pots and grown to maturity in a greenhouse. Tubers from each transformed plant line were collected and stored at 4°C for 3 months. A sample of 4 tubers from each transformed event was sliced from apical to basal end, and two 1/16-inch (0.4 cm) slices from each tuber were fried at 185°C (365 °F) for approximately 150 s. Chip color determination was made visually on the chip sample from each event with the use of the Potato Chip Color Reference Standards developed by Snack International (previously Snack Food Association), Cleveland, Ohio. Kal91.3 had the best chip color of the different events and was selected for advanced molecular analysis.

### 2.4. Insert Copy Number and Characterization

Droplet digital PCR (ddPCR) is a quantitative endpoint assay used to measure the T-DNA insert copy number in Kal91.3. ddPCR was used to test insertion number by quantifying the *nptII* gene target across Kal91.3 relative to a native potato amino‑acid permease gene *StAAP2*. The results were compared to the host variety Kalkaska. The ddPCR procedure described in Collier et al.^22^ was conducted on Kalkaska and Kal91.3 genomic DNA.

The Kal91.3 T-DNA sequence data were generated first by utilizing Xdrop™ enrichment technology and second by Sanger sequencing technology. The utilization of Xdrop™ technology for the characterization of T-DNA can be reviewed in our recent publication.^23^ The Xdrop™ enriched DNA was then subjected to debranching, treated with T7 Endonuclease I, and followed by size selection, end-repair, barcoding, and adaptor ligation using the Oxford Nanopore Technology (ONT) Ligation Sequencing Kit. After library generation, the sample was loaded onto a GridION flow cell R9.4.1 (20 fmol), as recommended by the manufacturer (Oxford Nanopore Inc.), to generate long-read sequencing data.

A more detailed description of the bioinformatics methods can be reviewed in Zarka et al.^23^ Briefly, the Kal91.3 bioinformatic analysis is as follows. To obtain sequences with high accuracy, the generated raw data files were base-called using Guppy 5.0.17 with super high accuracy and quality 10 filtering settings. Using the sequence data and the potato reference genome *Solanum tuberosum* DM1-3 PGSC v4.04 pseudomolecules downloaded from http://spuddb.uga.edu/index.shtml,^24^ the T-DNA was mapped to the genome. After masking those regions, a new reference genome was created by adding the T-DNA only region of the pINVBP1 sequence as an extra chromosome. All reads were then mapped to the new masked genome containing the T-DNA. The sequence viewer Integrative Genomics Viewer (IGV) was used to examine the coverage profile for the T-DNA. Both primary and supplementary mapping reads that map to the T-DNA were extracted; these are the reads of interest for finding insertions. Using the reference and the extracted reads from the T-DNA, a list of possible insertion areas was generated with coverage in the bam file. The IGV viewer was then used to identify the insertion borders.

### 2.5. Junction and Flanking Sequencing Analysis

Flanking sequence analysis includes PCR amplification of the T-DNA/chromosomal junctions for each border (left and right). Sanger sequencing was used to obtain 500 bp of T-DNA at each border, as well as 1000 bp of chromosomal DNA, and each of the borders. Since there are two T-DNA inserts in Kal91.3, both inserts were analyzed. Forward and reverse primers were designed for each insert (Chr 1 and Chr 3) and for each T-DNA border. DNA was isolated from fresh plant leaf tissue using Qiagen DNeasy Plant Mini Kit Cat No./ID: 69104 (Qiagen, Georgetown, Maryland) according to the manufacturer’s instructions. PCR analysis and gel electrophoresis were performed to obtain PCR amplicons for use in Sanger sequencing. Six primer sets were used to obtain amplicons containing at least 1000 bp of flanking sequence and 500 bp of T-DNA sequence for each insert and each border. Supplementary Table S2 lists the primers, sequence, annealing temperatures, and length of the amplicon. Each PCR was performed using the following conditions: 95°C for 3 min, then 35 cycles at 95°C for 30 s, (annealing temp.; see Supplementary Table S2) 30 s and 72°C 2 min, then held at 4°C. The PCR samples were then electrophoresed on 0.8% agarose gel using a 1-kb standard (STD) from (NEB 1-kb N3232S, New England Biolabs, USA). Qiaquick Gel Extraction Kit Cat no. / ID:28704 (Qiagen) was used to purify the PCR fragments before Sanger sequencing.

### 2.6. Absence of Backbone Analysis

Agrobacterium-mediated transformation can result in transfer of plasmid backbone DNA, in addition to the intended T-DNA.^25^ The absence of pINVBP1 backbone in Kal91.3 was confirmed through two assays. PCR analysis with six primer sets that span the entire pINVBP1 plasmid backbone was conducted to show the absence of large plasmid backbone sequences, and Southern analysis, using six PCR‑derived labeled probes that span the entire pINVBP1 backbone region, was performed to detect small backbone sequences.

DNA primers and probes that span the backbone were developed and used to analyze Kal91.3 for large backbone sequences (primers for PCR analysis) and small backbone sequences (probes for Southern analysis). The DNA primers and the location of each primer in plasmid pINVBP1 can be viewed in Supplementary Table S3. These primers were then used for either PCR analysis or for Southern probe development.

PCR analysis with the six primer sets that span the entire pINVBP1 plasmid backbone was conducted to scan for large plasmid backbone sequences. A unique primer set for the Kal91.3 event was used as a positive control. Kal91.3 FWD primer: 5’CGAGCCGGAAGCATAAAGTGTAAAG3’ and Kal91.3 REV primer: 5’TTCAATGGGATTGGGAGATACGTC3.’ The primer set results in a band at 1049 bp if the DNA is positive for the Kal91.3 event. The six primer sets and the unique primer set PCRs were performed using the following conditions: 95°C for 3 min, then 35 cycles at 95°C for 30 s, 58°C for 30 s, and 72°C for 2 min. The PCR samples were then electrophoresed on a 0.8% agarose gel using a 1-kb standard (STD) from NEB 1-kb N3232S, New England Biolabs, USA. Using the PCR conditions described above, probes for Southern analysis were prepared with a Roche digoxigenin (DIG)-labeled random-primed probe labeling kit (MilliporeSigma, Burlington, MA) according to the manufacturer’s instructions.

Plant DNA isolation was completed according to Saghai-Maroof et al.^26^ with young potato leaves. DNA concentration was measured with the Quant -iT^TM^ PicoGreen^TM^ dsDNA Assay Kit (ThermoFisher, Waltham, MA), and quality was confirmed by running the DNA on a 0.8% agarose gel in 1X Tris/Acetate/EDTA (TAE) for 30–40 min at 80 V. For each sample, 20 μg of DNA was digested overnight in a 40 μl final volume with 3 μl (10 units/μl) *HindIII* restriction enzyme (New England Biolabs, Ipswich, MA) and 1x rCutSmart buffer at 37°C for 12 hrs. For positive controls, the plasmid pINVBP1 was linearized with *HindIII* using 1x rCutSmart buffer at 37°C for 12 hrs. The digested plasmid DNA was at a concentration of 2 ng/μl and 0.3 μl was used as a positive control or spiked into a lane within the Kalkaska control chromosomal DNA.

Digested DNA described above was mixed with Gel Loading Dye, Purple (New England Biolabs, Ipswich, MA), loaded onto large 0.8% agarose gels containing 0.5X TBE buffer and 3–5 μl SYBER Safe (10 mg/ml) and run at 25 V for 18 hrs. DIG-labeled standard was run on the gel. 2 µl was used in each lane. The transfer of DNA to the nylon membrane (Hybond N, Amersham) was carried out as described by Douches et al.^21^ The nylon membrane carrying the transferred DNA was prehybridized in 25 ml/blot pre-warmed hybridization solution, and hybridization was completed as described by Douches et al.^21^ The detection reaction was carried out with 2 ml of ready-to-use CDP-Star solution for each blot, according to the manufacturer’s instructions (Roche, Basel, Switzerland). The membrane was then detected using a BioRAD Chemi-Doc MP Imaging System (Hercules, CA).

### 2.7. Stability of Insert Over Clonal Cycles

Commercial potatoes are propagated vegetatively and do not undergo meiotic recombination, therefore, potatoes are expected to be genetically stable. T-DNA PCR analysis specific to the Kal91.3 event was conducted over three vegetative propagation cycles to assess the stability of the intended genetic change. DNA insert stability was demonstrated by extracting and evaluating DNA from generation-3 (G3) samples through PCR analysis. For G3 analyses, 3 tubers from 3 plants from Kal91.3 and a plant from the non-transgenic control, Kalkaska, that sustained 3 tuber cycles (1 in the greenhouse and 2 in field trials), were collected from MSU Montcalm Research Center, 4629 W. McBrides Road, Lakeview, MI 48,850, field‑trial site during the 2023 field season. Genomic DNA was isolated using Qiagen DNeasy Plant Mini Kit Cat No./ID: 69,104, according to the manufacturer’s instructions. The stability of the inserted T-DNA in the events is shown by PCR analysis using primers that are unique to the Kal91.3 event and unique to each insert. These primer sets (Supplementary Table S4) were designed with one primer located in the T-DNA and the other primer located in the chromosomal location for each of the two inserts in Kal91.3. PCR was performed using the following conditions: 95 °C for 3  min, then 35 cycles at 95 °C for 30  sec, 58 °C for 30  sec, 72 °C for 1  min 20 sec. The PCR samples were then electrophoresed on a 0.8% agarose gel using a 1 kb standard (STD) from (NEB 1000 bp, New England Biolabs, USA.

### 2.8. Open Reading Frame, Allergenicity and Toxicity

The NCBI Open Reading Frame (ORF) Finder algorithm (https://www.ncbi.nlm.nih.gov/orffinder/) was used to identify all ORFs. The analysis for the Kal91.3 event was performed on each of the two inserts by examining 1000 bp of the chromosomal flanking sequence and 500 bp of the T-DNA region for each of the left T-DNA border region and the right T-DNA border region. The ORF Finder was then used to search for known protein matches using NCBI’s SmartBLAST/BLASTP. The parameters for the ORF search included a minimum length of 30 codons, the standard genetic code and the use of “ATG” as the start codon. Any open reading frames (ORFs) longer than 80aa were compared with protein translations of known allergens using the following: Allergenicity: bioinformatics analyses were completed using the Allergen Online (AllergenOnline Database v23 (January 30, 2025), http://www.allergenonline.org/databasefasta.shtml) and COMPARE database (https://comparefasta.comparedatabase.org/index.php). Toxicology bioinformatics analyses were completed using NCBI BLAST-P and UniProtKB/Swiss-Prot database (https://blast.ncbi.nlm.nih.gov/Blast.cgi)

### 2.9. RNA Expression Analysis

Kalkaska and Kal91.3 field-grown tuber seed-pieces were used as planting material at the MSU Montcalm Research Center field trial site during the 2023 field season. The trial was planted in May. Leaf tissue was collected in early September and used fresh. Tubers were harvested in late September and subsequently stored in the dark at room temperature for 2 weeks. Total RNA was isolated from leaf and tuber tissue by using the Qiagen RNeasy Plant Kit. Cat. No. 74904 (Qiagen, Hilden, Germany) according to the manufacturer’s instructions to obtain 15–20 μg.

Total RNA extracted from the different samples, was analyzed using the NanoString nCounter® analysis system^27^ and the gene expression Elements XT TagSet-12 which targeted the *VInv* gene and the *nptII* marker gene. Levels of the *VInv* gene and the introduced *nptII* marker gene were measured, along with a range of internal control genes. A range of internal controls was used to identify at least one control in the same expression range as the introduced genes, such that normalization could be done using equivalent signal levels. Internal control genes included: Actin, elongation factor 1-alpha, Cox1-B, potato vacuolar invertase, potato UDPG-glycosyltransferase, and potato sucrose-phosphate synthase 2. Actin was used for normalization.

Analyses of differential expression was performed using nCounter® with Elements™ XT Reagents according to the manufacturer’s specifications. A multiplexed probe library (nCounter® elements CodeSet) was designed with two sequence-specific probes for each gene of interest. The probe design was provided by NanoString as part of the kit purchase. DNA probes were then synthesized from Integrated DNA Technologies (IDT), Coralville, Iowa, USA. The probes used are described in Supplementary Table S5. Probes were mixed with 100 ng of purified total RNA and hybridized according to the manufacturer’s instructions. Samples were loaded on an nCounter® SPRINT^™^ Cartridge and processed with an nCounter® SPRINT^™^ Profiler (NanoString Technologies, USA) to quantify the transcripts. The nCounter raw expression data file (RCC) obtained was uploaded into the nSolver Analysis Software 4.0 for review of quality control metrics. Biological triplicates were analyzed.

### 2.10. Expressed Protein Analysis

Kalkaska and Kal91.3 field-grown tuber seed-pieces (G2) were used as planting material at the MSU Montcalm Research Center field trial site during the 2023 field season. Leaf tissue was collected in September and used fresh. Tubers were harvested in September and subsequently stored in the dark at room temperature for 2 weeks.

Levels of NPTII in leaves and tubers, were determined using a quantitative ELISA kit (PSP 73,000, Agdia, Elkhart, IN, USA) specific for NPTII. Additionally, a quantified NPTII standard (LST 73,000, Agdia, Elkhart, IN, USA) was purchased to use in creating standard curves. Tissues were homogenized in ratios of 10:1 (NPTII) volume extraction buffer/fresh leaf weight or 10:1 (NPTII) volume extraction buffer/fresh tuber weight in disposable plastic grinding bags (Agdia, Elkhart, IN) using a mortar. For the purpose of generating a standard curve, NPTII standard was used at concentrations of 18, 9, 4.5, 2.25, and 1.125 ng/mL. Both standard and homogenate preparations were loaded onto the microtitre plate in biologically triplicate wells (100 μL in each well) and incubated for 2 hr at room temperature. The assay was then completed according to the kit instructions. The absorbance of the reaction product was measured at 650 nm using a BioTek Synergy HT microplate spectrophotometer (Bio-Tek Instruments, Winooski, VT, USA).

### 2.11. Trait Efficacy and Compositional Assessments for Kal91.3

Field-grown tuber seed pieces were used as planting material at the MSU Montcalm Research Center field‑trial site during the 2023 and 2024 field seasons. The cultivation practices, including soil preparation, fertilizer application, irrigation, and pesticide-based control methods, according to MSU production recommendations.^18^

Field trials were planted using a randomized complete block design (RCBD). Four blocks (replicates) at each site included plots of test and control varieties as treatments. Each plot contained four rows. The rows were 6.1 m (20 ft) long, and seed spacing was one tuber every 28 cm (11 in). The seed tubers were placed by hand to a depth of approximately 10.6 cm (4 in).

To assess the trait efficacy of Kal91.3, potato tubers from both Kalkaska and Kal91.3 were harvested from the field trials and stored at 4°C (40°F) for 6 months and 8 months, respectively. Standard chip processing was then directly conducted on random samples from the cold‑stored tubers of Kalkaska and Kal91.3.

Compositional studies, sugar analysis and glycoalkaloids analysis were conducted on Kal91.3 and Kalkaska. The analysis examined to examine the nutritional composition, sugar content, and glycoalkaloids using freshly harvested tubers, not tubers stored in cold. Fresh tubers were used to show that Kal91.3 nutrition remains within the normal levels for potatoes compared to Kalkaska and conventional potatoes. The 2023 field samples included two randomly selected tubers from each of the four replicates, and the 2024 samples contained three randomly selected tubers also from each of the four replicates. Tubers were shipped to Eurofins (Wilson, NC) for analysis. A fact sheet from Eurofins describing the analysis methods and parameters (Eurofins PQDL Nutritional Facts 8–25-25) is included in the Supplementary Information, Figure S1.

Total glycoalkaloids (TGA) were reported as the sum of α-solanine and α-chaconine. TGA extraction by cutting opposite eighths of the tuber, dicing them with a vegetable dicer, and freeze-drying the subsequent tissue. The sample was then ground into a homogeneous dry mixture. For analysis of total tuber glycoalkaloids, ground, freeze-dried tissue from six randomly selected tubers, from the 2024 trial, were selected and sent to USDA ARS (E. Grandforks, MN) for analysis. The extraction procedure was completed as described in Edwards and Cobb^28^ and Mweetwa et al.^29^ TGAs were fractionated by HPLC (Agilent HP 1200 Series, Santa Clara, CA) on a C18 reversed-phase column (Agilent Eclipse XDB[1]C18, 5.0 μm pore size and 4.6 150 mm). TGAs were eluted with monitoring by diode array detection between wavelengths 190 to 240 nm. Quantification of TGAs was based on peak absorbance area at a wavelength of 202 nm.

## 3. Results

### 3.1. Molecular Characterization of Kal91.3

The T-DNA insert copy number, structure, flanking sequences, genomic integration site, and the absence of backbone DNA were characterized in the Kal91.3 potato line. The data and analysis are described in the sections below. This was submitted as part of a Regulatory Status Review (RSR) through the USDA following the guidelines^19^ and to the FDA as a Biotechnology Notification File (BNF000205) through the FDA’s Biotechnology Final Consultation Food Safety Evaluation for New Plant Variety Consultations^20^. The outcomes of these submissions are described in the discussion section.

**Figure 2.**
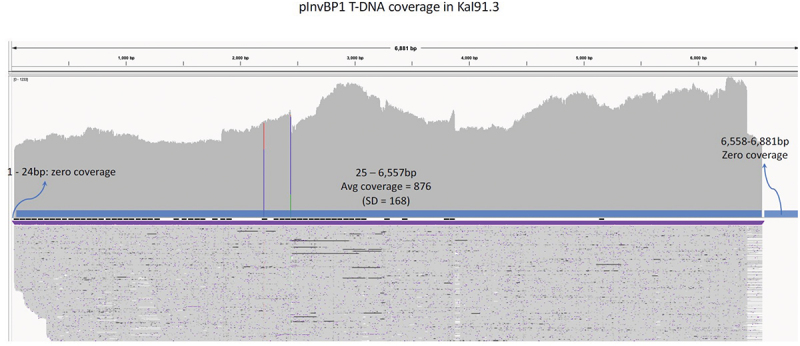
Summary of sequence read alignments to the pINVBP1 T-DNA.

The ddPCR method was used to determine the insertion number by quantifying the *nptII* gene target across Kal91.3 relative to a native potato amino acid permease gene *StAAP2*. The results were compared to the host variety Kalkaska. The ratio (*nptII: StAAP2*) for Kalkaska was 0.2, and Kal91.3 was 2.2, indicating that there are two copies of the *nptII* gene and two T-DNA inserts. This was further analyzed and confirmed by characterizing the insert location with sequence analysis, as described in the section below. The insert copy number was corroborated with the use of Xdrop™ enrichment technology (Samplix, Denmark) and by utilizing Nanopore sequencing followed by Sanger sequencing. The sequence data also indicated that the two copies of the T-DNA were inserted at separate loci, which were identified as being on chromosome 1 and chromosome 3. A significant depth of coverage across the T-DNA was achieved, see Figure 2. The two inserts combined had an average of 876X coverage across the T-DNA. Junction-finding scripts using these sequences and the DM potato reference genome^30,31^ specifically PGSC *S. tuberosum* group *Phureja* clone DM1-3 pseudomolecules (v4.04), indicated two insertion sites, corroborating T-DNA copy number by ddPCR. Note in Figure 2 that the T-DNA border segments show zero coverage. These are the border regions that were deleted in both T-DNA insertions.

Sequenced reads mapping to the pINVBP1 T-DNA insert are summarized by plotting depth of coverage. The left side of the figure is the RB (at 1124 of pINVBP1) and the right side shows the LB (at 7979 of pINVBP1). The two inserts combined had an average coverage of 876× across the T-DNA. The T-DNA border segments in the figure have a section with zero coverage. These are the border regions that were deleted in both T-DNA insertions.

Further analysis of the sequence data showed that one insert is located on chromosome 1 at 6,820,002 and 6,820,965 with a 963 bp chromosomal deletion compared to the PGSC *S. tuberosum* group *Phureja* clone DM1-3 pseudomolecules (v4.04) reference sequence. This insert has a truncation of 454 bp of the left T-DNA region (bases 7549 through 8003 of the pINVBP1 T-DNA (Supplementary Table S1 Genetic Elements of the DNA Insert of pINVBP1), subsequently deleting the NOS terminator of the *nptII* selection marker gene. The second insert is located on chromosome 3 at 55,608,167 and 55,608,288 with a 121 bp chromosomal deletion. The insert also has a truncation of 323 bp of the left T-DNA region (bases 7680 through 8003 of the pINVBP1 T-DNA, deleting part of the NOS terminator of the *nptII* selection marker gene.

**Figure 3.**
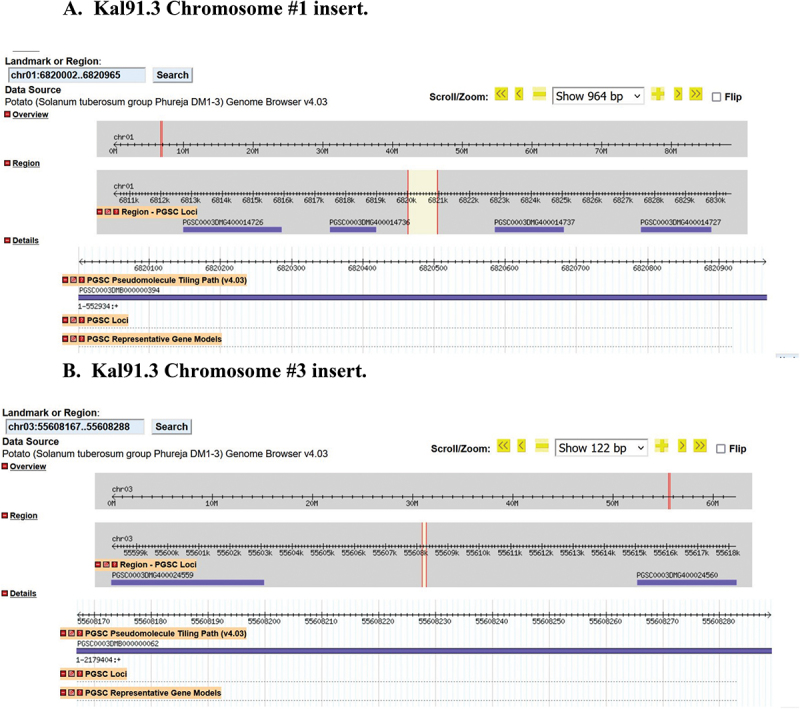
Location of the pINVBP1 T-DNA inserts located within (A) chromosome #1 and (B) chromosome #3.

Although both inserts have chromosomal deletions, 963 bp for the chromosome 1 T-DNA insert and 121 bp for the chromosomal 3 T-DNA insert, they do not interrupt or delete any chromosomal genes when compared with the DM1-3 pseudomolecules (v4.04) reference sequence (Figure 3). The truncations of left T-DNA, which include 454 bp in chromosome 1 and 454 bp and 323 bp in chromosome 3, both involve the deletion of all or most of the NOS terminator for the *nptII* marker gene. Since the *nptII* gene is only used in the selection of the line following transformation, its expression does not impact the vacuolar acid invertase silencing of the Kal91.3 line. A summary of the Kal91.3 T-DNA inserts is shown in Table 1.

Graphic of the results from inserting the pINVBP1 T-DNA insert chromosomal location coordinates within PGSC*S. tuberosum*group*Phureja*clone DM1-3 pseudomolecules (v4.04) reference sequence database. A. Chromosome #1 insert has a 963 bp region that was deleted from the genome during T-DNA insertion (yellow with red borders) and its identity to the reference genome (grey highlight) shows the deleted gap and that the insert is not interrupting any genes. B. Chromosome #3 insert has a 121 bp region that was deleted from the genome during T-DNA insertion (yellow with red borders), and its identity to the reference genome (grey highlight) shows the deleted gap and that the insert is not interrupting any genes.

**Table 1.** Summary of the Kal91.3 T-DNA inserts, T-DNA deletions, location in the T-DNA and impact.

T-DNA Insert in Kal91.3	Deletion of T-DNA	Location in pInvBP1	Function of Deleted DNA	Impact of Deleted DNA for line
T-DNA Insert Chr.1	NOS terminator for the *nptII* gene was deleted	7549–8003 (454 bp)	The NOS terminator defines the end of the transcription of the *nptII* gene for Kanamycin resistance.	No impact. NPTII was only used as a selectable marker during the initial transformation of the line
T-DNA Insert Chr.3	Part of the NOS terminator for the *nptII* gene was deleted	7680–8003 (323 bp)	The NOS terminator defines the end of the transcription of the *nptII* gene for Kanamycin resistance.	No impact. NPTII was only used as a selectable marker during the initial transformation of the line

**Figure 4.**
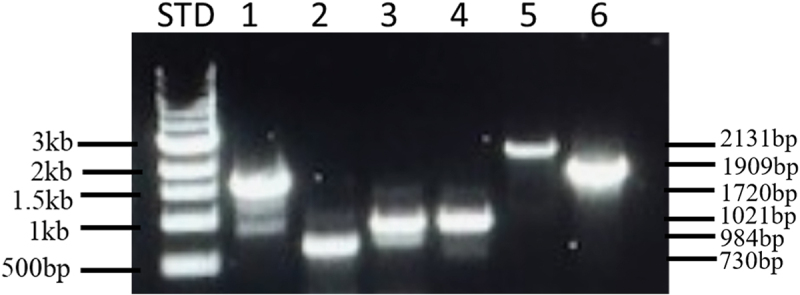
PCR flanking sequence analysis.

Flanking sequence analysis included PCR amplification of the T-DNA/chromosomal junctions for each border (left and right) and Sanger sequencing to obtain 500 bp of T-DNA at each insert and each border, as well as 1000 bp of chromosomal DNA for each insert and each border. Six primer sets were used to obtain amplicons (containing at least 1000 bp of flanking sequence and 500 bp of T-DNA sequence for each insert and each border). PCR and gel electrophoresis with primer sets 1 through 6 produced the expected amplicon sizes: the amplicon size (set 1: 1720 bp, set 2: 730 bp, set 3: 984 bp, set 4: 1021 bp, set 5: 2131 bp and set 6: 1909 bp) see Figure 4. These amplicons were then used for Sanger sequencing to characterize the flanking regions of each insert at the borders. Supplementary Table S6 provides 1 kb chromosomal and 500 bp T-DNA sequencing results for both the left and right border junctions for each insert.

Lane STD:standard (NEB 1kb N3232S, New England Biolabs, USA), lane 1–6:primer sets 1 through 6 that produced amplicons, which were then used for Sanger sequencing the flanking regions of each insert and the borders. The amplicon size for set 1(lane 1):1720 bp, set 2(lane 2):730 bp, set3(lane3):984 bp, set4(lane 4): 1021 bp, set 5(lane5):2131 bp and set 6(lane6):1909 bp.

**Figure 5.**
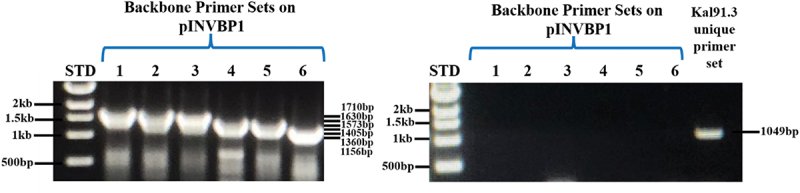
PCR large backbone analysis of pINVBP1 plasmid and Kal91.3.

The absence of the pINVBP1 backbone in Kal91.3 was confirmed by PCR analysis for screening of large backbone fragment inserts and Southern analysis for the detection of small backbone sequences. For large plasmid backbone analysis, primer set amplicon sizes of 1: 1710 bp, 2: 1630 bp, 3: 1573 bp, 4: 1405 bp, 5: 1360 bp, 6: 1156 bp were produced for the analysis using the pINVBP1 plasmid. When the same primers were used to test Kal91.3, no amplification was observed, indicating that no large insertions of the plasmid backbone occurred during the transformation process (Figure 5).

Lane STD: Standard (NEB 1kb N3232S, New England Biolabs, USA), lane 1–6: primer set amplicon sizes 1: 1710 bp, 2: 1630 bp, 3: 1573 bp, 4: 1405 bp, 5: 1360 bp, 6: 1156 bp when used with the plasmid pINVBP1 DNA (left side). However, since there are no large backbone fragments in Kal91.3 there was no amplification in sets 1–6 (right side). The Kal91.3 unique primer set (far right) was used as a positive control, producing the expected 1049 bp band.

**Figure 6.**
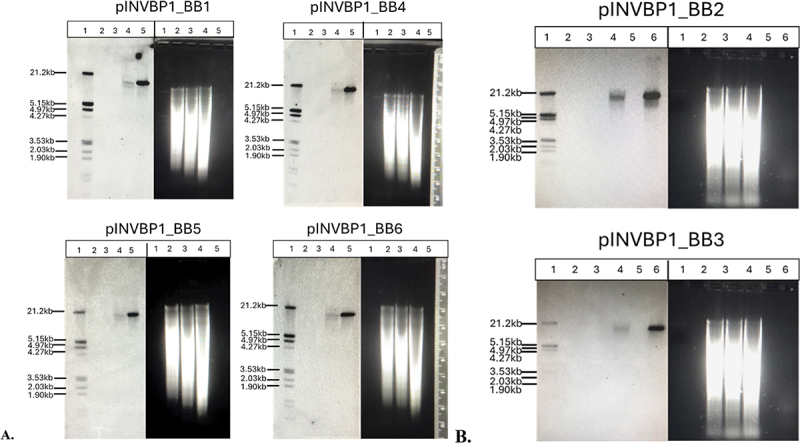
Small backbone Southern DNA analysis.

The small plasmid backbone results are shown in Figure 6 below. The linearized pINVBP1 plasmid and Kalkaska DNA spiked with the linearized pINVBP1 plasmid were used as positive controls. No small fragments were detected with the six different probes that spanned the entire plasmid pINVBP1 backbone. The control lanes containing the plasmid with the backbone were detectable with each probe. Likewise, the non-transgenic host Kalkaska DNA spiked with pINVBP1 plasmid lanes were also detectable with each probe. Southern analysis data shows that there are no small backbone DNA sequences of the plasmid present in line Kal91.3.

A. Southern blot andagarose gel U.V. image. Lane 1, DIG STD,lane 2 Kalkaska DNA HindIII,lane 3. Kal91.3 HindIII,lane 4 Kalkaska DNA HindIII spiked with pINVBP1 plasmid. Lane 5. pINVBP1 plasmid. The blot/gel on the top left was detected with probe BB1, top right BB4, bottom left BB5 and bottom right BB6. There are no small backbone fragments in Kal91.3 with these probes. The plasmid containing the backbone and the Kalkaska spiked with the plasmid gave positive results. B. Southern blot and Agarose gel U.V. images. Lane 1, DIG STD, Lane 2 Kalkaska DNA HindIII, Lane 3. Kal91.3 HindIII, Lane 4 Kalkaska DNA HindIII spiked with pINVBP1 plasmid. Lane 5. BLANK, Lane 6. pINVBP1 plasmid. The blot/gel on the top was detected with probe BB2, top right BB4 and the bottom with BB3. There are no small backbone fragments in Kal91.3 with these probes. The plasmid which contains the backbone and the Kalkaska spiked with the plasmid gave positive results.

### 3.2. Stability of Insert Over Clonal Cycles

**Figure 7.**
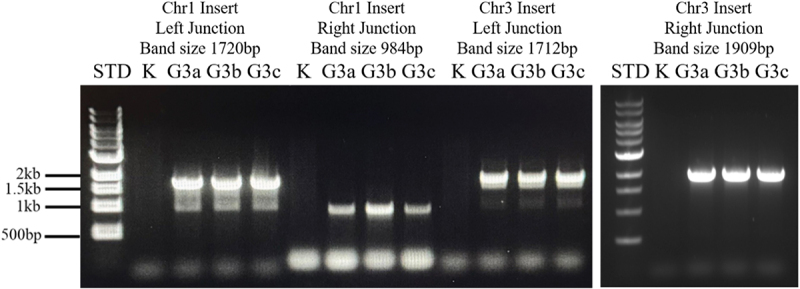
Stability of the insert over clonal cycles: PCR analysis.

The results, observed in Figure 7, confirm that the T-DNA from line Kal91.3 is stably inserted into each line. The correct amplicon bands are present in the three replications of Generation 3 tubers for each of the 4 unique T-DNA border site primer sets tested. These results confirm stability across clonal cycles for the Kal91.3 line.

PCR analysis of Kal91.3 line generation 3 tubers using 4 unique T-DNA junction primer sets. This figure shows the results of PCR analysis using junction primer sets for each border of each of the two T-DNA inserts located in chromosomes 1 and 3. The 3 replicated samples of the Kal91.3 generation 3 are represented as G3a, G3b, and G3c. The PCR set 1 (see Supplementary Table S4) was used for Chr1 insert left junction with a 1720 bp band present. The PCR set 3 was used for Chr1 insert right junction with a 984 bp band present. The PCR set 5 was used for Chr3 insert left junction, with a 1712 bp band present. The PCR set 6 was used for Chr3 insert right junction, with a 1909 bp band present.

### 3.3. Open Reading Frame, Allergenicity and Toxicity

#### 3.3.1. Kal91.3 Chromosome 1 Border Analysis

The ORF left border analysis showed that there are 3 significant ORFs created with 80aa or more. ORF#1 is located within the T-DNA region and was identified as 80% identical to D-nopaline dehydrogenase [*Agrobacterium tumefaciens* (strain T37)], which is part of the terminator region of the NPTII cassette. ORF #2: 38% identity to 3-phosphoshikimate 1-carboxyvinyltransferase, and ORF #3: 42% identity to PWWP domain-containing DNA repair factor 3A were also identified. The ORF right border analysis showed that there was 1 significant ORFs created with 80aa or more, however, it had only a 36% identity to LRR receptor-like serine/threonine-protein kinase GSO2. The protein sequences in the 4 ORFs were further analyzed, and there were no significant matches to allergens and no matches with up to 8mer allergen motifs. Toxicology bioinformatics analyses revealed no matches to known toxins.

#### 3.3.2. Kal91.3 Chromosome 3 Border Analysis

The left border ORF analysis shows that there was 1 significant ORF created with over 80aa. As in the other insert in CHR1 on the left border, this insert also has the ORF identified as 93% of the D-nopaline dehydrogenase [Agrobacterium tumefaciens (strain T37)], which is part of the terminator region of the NPTII cassette. The right border ORF analysis showed that there was 1 significant ORF created with 80aa or more, however, it only had a 36% identity to a Translocon at the inner envelope membrane of chloroplasts 214. The two ORF protein sequences were analyzed further using COMPARE and Allergen Online, and there were no significant matches to allergens and no matches with up to 8mer allergen motifs. Toxicology bioinformatics analyses revealed no matches to known toxins.

A summary of the ORF, allergy and toxicity assessment is displayed in Table 2. The results indicate that both T-DNA inserts in Kal91.3 did not result in unintended ORFs that have any allergenic or toxic properties.

**Table 2.** Summary of ORF, allergy and Toxicity assessment in Kal91.3.

Insert Location	T-DNA Border	ORF#	Significant Match / Identity	Allergenicity Assessment COMPARE and Allergen Online	Toxicity Assessment NCBI BLAST-P and UniProtKB/Swiss-Prot database
Kal91.3 Chr.1	Left	ORF #1	80% identity to D-nopaline dehydrogenase, within T-DNA region, part of the NPTII cassette terminator	No significant allergen matches or 8-mer allergen motifs.	No matches to toxins
Kal91.3 Chr.1	Left	ORF #2	38% identity to 3-phosphoshikimate 1-carboxyvinyltransferase	No significant allergen matches or 8-mer allergen motifs.	No matches to toxins
Kal91.3 Chr. 1	Left	ORF #3	42% identity to PWWP domain-containing DNA repair factor 3A	No significant allergen matches or 8-mer allergen motifs.	No matches to toxins
Kal91.3 Chr. 1	Right	ORF #1	36% identity to LRR receptor-like serine/threonine-protein kinase GSO2	No significant allergen matches or 8-mer allergen motifs.	No matches to toxins
Kal91.3 Chr. 3	Left	ORF #1	93% identity to D-nopaline dehydrogenase (*Agrobacterium tumefaciens* strain T37); within T-DNA, part of the NPTII cassette terminator region	No significant allergen matches or 8-mer allergen motifs.	No matches to toxins
Kal91.3 Chr.3	Right	ORF #1	36% identity to Translocon at the inner envelope membrane of chloroplasts 214 (TIC214)	No significant allergen matches or 8-mer allergen motifs.	No matches to toxins

### 3.4. Characterization of mRNA in Kal91.3

**Figure 8.**
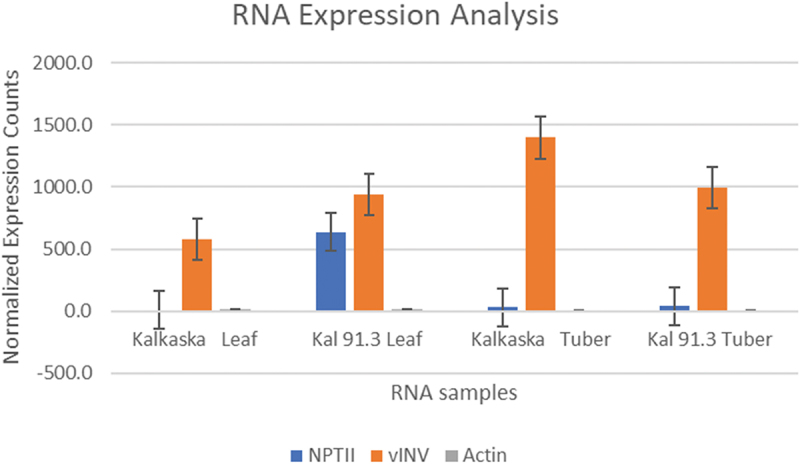
RNA expression analysis of *VInv* and *nptII* in Kal91.3. the *VInv* and *nptII* mRNA of leaf and tuber tissue from both Kalkaska and Kal91.3 were analyzed using actin as the normalized control. The graph shows a comparison of the levels of *nptII, VInv* and *actin* in leaf tissue and tuber tissue of both Kalkaska and Kal91.3 replicated samples.

Lower levels of reducing sugars were achieved by downregulating potato *VInv* in Kal91.3. Transcript levels in Kal91.3 were compared to transcript levels in Kalkaska to confirm reduced transcript levels of *VInv*. Transcript levels of the introduced *nptII* marker gene were measured at the same time in the same samples. The results of the mRNA analysis showed that Kal91.3 had reduced expression of *VInv* RNA in tubers compared to the Kalkaska control (Figure 8). The *nptII* RNA expression in the tubers of Kal91.3 is at a very low level of detection.

The pInvBP1 T-DNA encodes neomycin phosphotransferase II, which confers resistance to the antibiotics neomycin and kanamycin and serves as a selectable marker for plant transformation. To show that the expression of NPTII falls within or below the previously evaluated levels, the NPTII levels in the leaf and tuber of Kal91.3 were analyzed. The Kal91.3 had 3 μg/g of NPTII expressed in the leaf tissue and 0.6 μg/g of NPTII in the tuber tissue. The NPTII protein expression levels in Table 3 show that they are at the same levels and at lower levels than those observed in previously released GM potatoes with NPTII. The Kalkaska had levels equal to the background.

**Table 3.** Levels of NptII present in Kal91.3 as determined by quantitative ELISA.

	REp1	Rep2	Rep3	Mean	NPTII well	Amount of tissue	μg NPTII/gram tissue
Kalkaska Leaf	0.428	0.46	0.447	0.445	<LOD	100 mg	
Kal91.3 Leaf	1.745	1.712	1.679	1.712	30 ng/ml	100 mg	**3.0 μg/g**
Kalkaska Tuber	0.422	0.475	0.497	0.465	<LOD	100 mg	
Kal91.3 Tuber	0.699	0.76	0.749	0.736	6 ng/ml	100 mg	**0.6 μg/g**

### 3.5. Trait Efficacy Assessment of Kal91.3 After Storage

**Figure 9.**
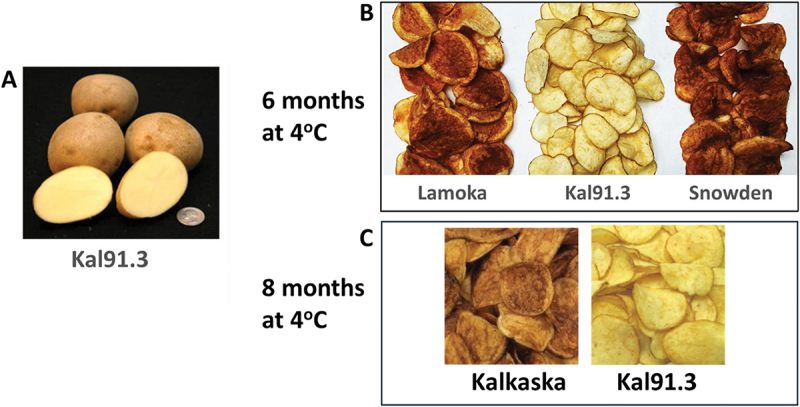
Trait efficacy of Kal91.3 after Storage.

To assess the trait efficacy of Kal91.3, potato tubers from both Kalkaska and Kal91.3 were harvested from the field trials, stored at 4°C for 8 months, and chip‑processed as described in the methods section. The results clearly showed Kal91.3 has significantly lower levels of reducing sugars (chips are light in color) compared to Kalkaska (chips are dark in color) (Figure 9). For comparison, Lamoka and Snowden, both commonly used commercial varieties, were stored along with Kal91.3 at 4°C for 6 months, and chip processed. Figure 9 shows that Kal91.3 chips were light in color and Lamoka and Snowden had very dark colored chips. These results can be attributed to the downregulation of vacuolar invertase in Kal91.3. The changes to levels of reducing sugars are not nutritionally consequential as they do not affect the levels of key nutrients important for potato.^32^

A. Fresh Kal91.3 potato tubers, whole and cut. B. Comparison of Kal91.3 and commercially used Lamoka and Snowden varieties processed into chips directly from 6months of storage at 4°C. C. Kalkaska and Kal91.3 potato chips processed directly from 8months storage at 4°C.

### 3.6. Compositional Analysis for Kal91.3

Compositional studies were conducted on freshly harvested Kal91.3 and Kalkaska tubers to show nutritional equivalence. All results are reported in fresh weight. Compositional assessments were performed for the following:

- Proximates, vitamins, minerals, and carbohydrates (Table 4);

**Table 4.** Proximates, vitamins, minerals, and carbohydrates in Kal91.3 tubers.

Variable	Variety	Mean	*p*-Value	N	Range		Combined Literature Range^1^	
					Min	Max	Min	Max
Protein (%)	Kal91.3	2.3	0.847	5	2.06	2.5	1.22	4.02
	Kalkaska	2.276		5	2.00	2.5		
Total Fat (%)	Kal91.3	0.136	0.271	5	0.1	0.22	0.100	0.740
	Kalkaska	0.108		5	0.1	0.14		
Ash (%)	Kal91.3	1.048	0.214	5	1.01	1.10	0.150	2.00
	Kalkaska	1.004		5	0.94	1.09		
Total Dietary Fiber (%)	Kal91.3	2.0	0.749	5	1.8	2.2	1.40	3.60
	Kalkaska	1.96		5	1.6	2.2		
Carbohydrates (%)	Kal91.3	16.06	0.377	5	12.02	19.77	3.68	25.1
	Kalkaska	14.19		5	8.71	17.4		
Moisture (%)	Kal91.3	80.5	0.356	5	76.6	84.9	71.8	85.6
	Kalkaska	82.5		5	79.0	87.7		
Total Vitamin D2 and D3 (IU/100 g)	Kal91.3	4	1.000	5	4	4	N/A	N/A
	Kalkaska	4		5	4	4		
Nitrogen – Combustion (%)	Kal91.3	0.368	0.841	5	0.4	0.4	N/A	N/A
	Kalkaska	0.364		5	0.32	0.33		
Potassium (mg/100 g)	Kal91.3	0.463	0.605	5	0.44	0.48	291	765
	Kalkaska	0.472		5	0.43	0.51		

^a^Combined literature ranges are from AFSI.^33^ Total dietary fiber range is from OECD.^32^
- Glycoalkaloids (Table 5).

**Table 5.** Glycoalkaloids in Kal91.3 and Kalkaska tubers.

Variable	Variety	Mean (mg/100 g)	*p*-Value	Standard Deviation	_N_	Range (mg/100 g)		Combined Literature Range (mg/100 g)^a^	
						Min	Max	Min	Max
Glycoalkaloids^b^	Kalkaska	12.5	NS^c^	1.3	2	10.86	14.13	3.20	210
	Kal91.3	11.8		1.3	2	9.06	14.56		

^a^Combined literature range from.^34^

^b^Total of α-solanine and α-chaconine.

^c^Means are not significantly different as determined by Tukey’s HSD (α = 0.05).

No statistical differences between Kal91.3 and Kalkaska were observed for proximates, vitamins, and minerals (Table 4). These results indicate that Kal91.3 was equivalent to Kalkaska for these analytes.

Glycoalkaloids are toxins commonly found in solanaceous crops, including potato, and 95% of the total glycoalkaloids in potato tubers consist of α-solanine and α-chaconine.^32^ The safety limit for total glycoalkaloids in tubers is 20 mg/100 g fresh weight.^35^ Total glycoalkaloids were reported as the sum of α-solanine and α-chaconine. There was no significant difference in glycoalkaloid levels between Kal91.3 and Kalkaska (Table 6). Both Kal91.3 and Kalkaska mean total glycoalkaloid concentrations were lower than the safety limit and fell within the combined literature range.^34^

**Table 6.** Sugar analysis of Kal91.3.

Variable	Variety	Mean (mg/100 g)	*p*-Value	*N*	Range (mg/100 g)		Combined Literature Range (mg/100 g)^2^	
					Min	Max	Min	Max
Glucose	Kal91.3	<15	0.1411	5	<15	<15	13	3,049
	Kalkaska	16		5	<15	17		
Fructose	Kal91.3	<15	N/A^a^	5	<15	<15	6.7	1,427
	Kalkaska	<15		5	<15	<15		
Sucrose	Kal91.3	42	0.0658	5	30	60	39.7	3,912
	Kalkaska	31		5	27	38		

^a^When all values are below the LOQ, standard deviation and ANOVA cannot be calculated and are reported as “NA”.

^b^Combined literature ranges from^33,36,37^

Sugars were analyzed from freshly harvested 2023 and 2024 field tuber samples. Fresh tubers were used to show that at harvest time, the Kal91.3 sugars are within the normal levels for potatoes compared to Kalkaska and conventional potatoes. The results show that mean levels of fructose, glucose, and sucrose of Kal91.3 tubers were within the combined literature range for conventional potatoes (Table 6). There were no significant differences between Kal91.3 and Kalkaska for fructose, glucose, or sucrose levels as determined by ANOVA (α = 0.05).

The nutrition assessment evaluating proximates, vitamins, minerals, and glycoalkaloids showed no statistical differences between values for Kal91.3 and Kalkaska when freshly harvested. Sugar analysis on cold-stored potatoes is not needed for a regulatory review because changes to levels of reducing sugars are not nutritionally consequential, as they do not affect the levels of key nutrients important for potatoes.^32^

## 4. Discussion

The development and analysis of Kal91.3 presented here is a comprehensive molecular, biochemical, and phenotypic characterization. The data demonstrate that RNAi-mediated suppression of vacuolar invertase was successfully achieved in this scab-resistant and high-performing potato background without introducing unintended compositional or genetic changes. Kal91.3 expresses the intended trait, resistance to cold-induced sweetening, greatly extending the potential length of its storage. All measured parameters indicated that Kal91.3 remained substantially equivalent to the non-modified parent variety, Kalkaska.

### 4.1. Kal91.3 Has Two Stable, Independent T-DNA Insertions

Initially, an estimate of copy number with ddPCR analysis revealed two T-DNA insertions. This was subsequently validated through Xdrop™ enrichment, Nanopore sequencing, and Sanger confirmation. The integration of two independent T-DNA copies could be an advantage with RNA silencing. *Solanum tuberosum* (potato) is a tetraploid and therefore has four copies of the invertase gene. The excellent chip quality after 8 months of cold storage may have been achieved due to a high level of gene silencing as observed by Bhaskar et al.^9^ The two insertion sites were fully characterized and, although small chromosomal deletions were observed in both, neither overlapped with annotated genes in the DM1-3 reference genome. These findings indicate that the two insertions are unlikely to have disrupted any endogenous gene function or phenotypic qualities of the Kalkaska host variety.

Both T-DNA copies in Kal91.3 involved truncations at the left T-DNA border, including partial or complete deletion of the NOS terminator associated with the *nptII* selectable marker cassette. Truncations of T-DNA are well-documented in Agrobacterium-mediated transformation,^38^ and because the silencing cassette, within the T-DNA of the Kal91.3 event, was intact, the truncations did not affect the intended RNAi function. Studies showing the absence of vector backbone sequences in Kal91.3, confirmed through both PCR and Southern blot analyses, support clean T-DNA integrations that meet regulatory expectations. The stability of the insertions across clonal generations was demonstrated by consistent amplification of all border junctions in multiple vegetative cycles. These results confirm the genetic integrity of Kal91.3, a requirement for commercial seed propagation.

### 4.2. No Unintentional Open Reading Frames, Allergens, or Toxins

Bioinformatic analyses of the inserted sequences and the junction regions revealed that no open reading frames were created that were similar to known allergens or toxins. The absence of unintended ORFs supports the conclusion that Kal91.3 does not pose new allergenic or toxicological risks.

### 4.3. Expression of NPTII is Low and Within Historical Safe Ranges

The *nptII* gene was used in the initial transformation procedure as a selectable marker for identifying potential transformed potato events. Protein expression analysis on Kal91.3, 3.0 μg/g in fresh leaf and 0.6 μg/g in fresh tuber tissue, confirmed that NPTII levels in the tubers and leaves fall within or below the range of previously released GM potatoes containing NPTII between 1995 and 2001. NPTII was detected in the tubers at 0.197 µg/g fresh weight (Russet Burbank NewLeaf®), and 0.5–2.9 µg/g fresh weight of the transgenic varieties Atlantic and Superior NewLeaf® (Decision document: DD1998-01 from the Canadian Food Inspection Agency). Other estimations of NPTII quantity in tubers were between 0.23 and 2.95 μg/g, according to the findings of Smith et al.^39^ A safety assessment of NPTII, was previously reported and found it safe for consumption.^40^ Globally, NPTII has had extensive regulatory evaluations and has a history of safe use in food, the environment, and other agricultural applications. The low expression levels in the analysis, found in Kal91.3, further supports its overall safety.

### 4.4. Suppression of Vacuolar Invertase and Intended Trait Performance

Kal91.3 RNA expression analysis demonstrated clear downregulation of *VInv* transcripts in tubers compared to the non-modified Kalkaska control. The reduction in transcript levels observed is consistent with the outcomes of RNA interference and the findings from other silencing studies in potato^9,12,13^ The suppression of *VInv* resulted in a significant reduction in reducing sugar accumulation during cold storage. After 6 months and again at 8 months at 4°C, Kal91.3 tubers produced light-colored chips, whereas Kalkaska tubers produced dark chips characteristic of CIS. These results support our understanding that the RNAi T-DNA within pINVBP1 prevents the conversion of sucrose into glucose and fructose during prolonged cold storage.

As described in the introduction, RNAi technology for invertase silencing has been commercially released by Simplot within their Innate potato products. Simplot used the same technology for its second-generation Innate 2.0 products and most recently within the third-generation Innate product BG25, FDA BNF 197^16^. In potatoes, a down-regulation of invertase, as seen with RNAi silencing, is a proven technology for commercial use.

### 4.5. Compositional Equivalence Demonstrated in Kal91.3

Across all measured proximates, vitamins, minerals, and sugars in Kal91.3 were statistically indistinguishable from those in the host Kalkaska variety. Additionally, the analysis of glycoalkaloid levels in both varieties was well below established safety thresholds and within the range reported for commercial potatoes. These Kal91.3 findings agree with the Codex Alimentarius framework for substantial equivalence, indicating that the genetic modification did not alter the nutritional profile of the potato crop.

### 4.6. RNAi-Based Trait Integration into a Commercial Conventionally-Bred Background

Kalkaska was conventionally bred for the highly valuable scab resistance trait, and suitability for chip processing. Our research showed no difference in yield, therefore, Kal91.3 maintained the high-yield trait of the host variety. Bhaskar et al.^9^ also described no yield loss. As described earlier, the use of the RNAi silencing technology allows for a downregulation of the invertase expression. In a recent research study conducted by Massa et al.^41^ they achieved a complete knockout of invertase in the Atlantic potato variety using CRISPR/Cas9 technology. The field trials of the Atlantic line, named PIRU INTA, showed a yield penalty of 56%. The authors describe the economic benefits even with the yield loss and suggest conducting larger field studies. In another CRISPR study, Zhu et al.^42^ utilized gene editing to delete a highly conserved 200-bp intronic enhancer (VInv In2En) within the potato *VInv* gene. They showed that expression of invertase in cold-stored tubers was significantly reduced in the lines with the deleted enhancer. Field studies on these gene-edited lines remain to be studied, but this offers a promising gene-edited approach.

Although further research with CRISPR technology is recommended, our current RNAi silencing is a successful commercial strategy. The incorporation of CIS resistance into the Kalkaska background represents a significant advancement for the potato industry. For a potato grower, being able to store tubers at low temperatures without compromising chip quality provides year-round processing capacity, supply chain stability, and waste reduction for the processing industry.

### 4.7. Regulatory Approval of Kal91.3 in the United States

On January 30^th^ 2024, the USDA APHIS completed a review of the Kal91.3 line, detailed in this manuscript, with RSR number 23–340-01rsr and responded by issuing non-regulated status.^43^ Kal91.3 is the first and currently the only public university and non-industry organization to obtain non-regulated status of a GM field-crop product from the USDA/APHIS. Following this approval, a biotechnology notification file (BNF) was completed and submitted to the Center for Food Safety and Nutrition (CFSAN) for the food safety evaluation of the Kal91.3 potato as part of FDA’s voluntary premarket consultation process in September of 2025. The primary intended use for Kal91.3 is human consumption in the form of fresh or processed potatoes, however a feed consultation with the Center for Veterinary Medicine (CVM), for Kal91.3, was also selected, in case potatoes, not used for processing or the fresh market, are fed to livestock. On March 25, 2026, the FDA concluded that the steps taken by the Michigan State University Potato Breeding and Genetics Program ensured that Kal91.3 complies with the legal and regulatory requirements that fall within FDA’s jurisdiction.^16^ Michigan State University is also the first public university to obtain a completed BNF consultation of a GM field crop product from the FDA. The Kal91.3 potato has full regulatory approval for production and consumption in the United States.

## 5. Conclusion

Biochemical and phenotypical analyses showed strong suppression of the *VInv* gene. An in-depth molecular characterization analysis revealed two stable independent T-DNA insertions that were free of plasmid backbone, no unexpected open reading frames, and no interruptions of existing chromosomal genes. The potato event Kal91.3 was developed and evaluated to demonstrate that vacuolar invertase was RNAi-suppressed stably in a conventionally bred potato without introducing unintentional changes to the host variety genome or composition.

The strengths of the Kalkaska variety, including scab resistance, high solids, and high agronomic performance, have been maintained. However, Kalkaska was prone to unpredictable accumulation of reducing sugars during storage. Kal91.3 will solve a significant problem in the potato chip processing industry. The high level of CIS, during long-term storage, results in light colored potato chips during processing. The improved chip quality of Kal91.3 is economical, environmentally sustainable, and benefits growers, processors, and consumers.

The thoroughness of the safety assessment of Kal91.3 is highlighted by the successful regulatory reviews and approval by both the USDA and the FDA. Kal91.3 has met all the safety requirements for commercial cultivation and use in food and feed. Kal91.3 is the first approved genetically modified field crop produced by a non-industry organization to receive full regulatory approval in the United States. This represents a milestone for public-sector breeding and biotechnology. In a broader scope, it demonstrates how conventional breeding can be combined with biotechnological techniques to overcome challenging constraints in the potato industry and serves as an example for future potato variety development.

## Supplementary Material

Supplemental Material

## Data Availability

The data presented in the study are deposited in the Dryad repository, accession number: DOI: 10.5061 at https://doi.org/10.5061/dryad.rjdfn2zsm
