# Supplementary material for: Development and regulatory approval of Kal91.3: combining advanced conventional breeding with genetic engineering to create a cold sweetening‑resistant chip‑processing potato
Source: GM Crops Food. 2026 Jul 28;17(1):2707844. doi: 10.1080/21645698.2026.2707844 (PMC13432818; doi:10.1080/21645698.2026.2707844)
Supplement: Supplemental Material [file KGMC_A_2707844_SM9188.docx]

**Supplementary Information**

**Supplementary Table S1. Genetic Elements of the DNA Insert of pINVBP1.**

An annotation of the pINVBP plasmid. The T-DNA Right Border (RB) site to Left Border (LB) highlighted in blue and the pINVBP1 plasmid backbone is in grey.

| **Acronym** | **Location**  **in pINVBP1** | **Size (bp)** | **Direction** | **Function** | **Origin** |
| --- | --- | --- | --- | --- | --- |
| **oriV** | **1..636** | **636** | **==** |  | pHELLSGATE  Sequence ID: AJ311874.1 |
| **Intervening**  **sequence** | **637..1123** | **486** |  | Used for cloning | pHELLSGATE |
| **RB T-DNA repeat** | **1124..1148** | **25** | **==** | T-DNA right border | *Agrobacterium tumefaciens* |
| **Intervening sequence** | **1149...1402** | **254** |  | Used for cloning | pHELLSGATE |
| **CAP binding site** | **1403..1424** | **23** | **==** | Used for cloning | pHELLSGATE |
| **Intervening sequence** | **1425..1438** | **14** |  | Used for cloning | pHELLSGATE |
| **lac promoter** | **1439..1472** | **32** | **=>** | Used for cloning | pHELLSGATE |
| **Lac operator** | **1473..1495** | **24** | **==** | Used for cloning | pHELLSGATE |
| **Intervening sequence** | **1496..1500** | **5** |  | Used for cloning | pHELLSGATE |
| **M13-rev** | **1501..1521** | **21** | **=>** | Used for cloning | pHELLSGATE |
| **Intervening sequence** | **1522..1526** | **5** |  | Used for cloning | pHELLSGATE |
| **SP6 promoter** | **1527..1553** | **27** | **=>** | Used for cloning | pHELLSGATE |
| **Intervening sequence** | **1554..2612** | **1059** |  |  | pHELLSGATE |
| **CaMV 35S promoter** | **2613..2958** | **347** | **=>** | 35S promoter | Cauliflower mosaic virus |
| **Intervening sequence** | **2959..2966** | **7** |  | Used for cloning | pHELLSGATE |
| **attB1** | **2967..2991** | **25** | **=>** |  | bacterial attachment site |
| **Intervening sequence** | **2992..3007** | **17** |  | Used for cloning | pHELLSGATE |
| ***VInv* (sense)** | **3008..3515** | **508** | **==** | Fragment of the acid invertase (sense orientation) | Generates double-stranded RNA that triggers the degradation of invertase transcripts |
| **Intervening sequence** | **3516..3535** | **21** |  | Used for cloning | pHELLSGATE |
| **attB2** | **3536..3560** | **25** | **<=** |  | bacterial attachment site |
| **Intervening sequence** | **3561..3586** | **26** |  | Used for cloning | pHELLSGATE |
| **PDK intron** | **3587..4355** | **769** | **==** |  | Pyruvate orthophosphate dikinase intron from Flaveria trinervia |
| **Intervening sequence** | **4356..4397** | **42** |  | Used for cloning | pHELLSGATE |
| **attB2** | **4398..4422** | **25** | **=>** |  | bacterial attachment site |
| **Intervening sequence** | **4423..4442** | **20** |  | Used for cloning | pHELLSGATE |
| ***VInv* (anti-sense)** | **4443..4950** | **508** | **==** | Fragment of the acid invertase (anti-sense  orientation) | Generates double-stranded RNA that triggers the degradation of invertase transcripts |
| **Intervening sequence** | **4951..4966** | **16** |  | Used for cloning | pHELLSGATE |
| **attB1** | **4967..4991** | **25** | **<=** |  | bacterial attachment site |
| **Intervening sequence** | **4992..4999** | **8** |  | Used for cloning | pHELLSGATE |
| **OCS terminator** | **5000..5707** | **708** | **=>** |  | pHELLSGATE |
| **Intervening sequence** | **5708..5747** | **41** |  | Used for cloning | pHELLSGATE |
| **SP6 promoter** | **5748..5766** | **19** | **<=** |  | pHELLSGATE |
| **Intervening sequence** | **5767..5794** | **28** |  | Used for cloning | pHELLSGATE |
| **Kozak sequence** | **5795..5804** | **10** | **==** |  | pHELLSGATE |
| **Intervening sequence** | **5805..5833** | **29** |  | Used for cloning | pHELLSGATE |
| **T7 promoter** | **5834..5860** | **29** | **<=** |  | pHELLSGATE |
| **M13-fwd** | **5860..5877** | **18** | **<=** |  | pHELLSGATE |
| **Intervening sequence** | **5878..5947** | **71** |  | Used for cloning | pHELLSGATE |
| **LacZ alpha** | **5948..6016** | **69** | **=>** |  | pHELLSGATE |
| **Intervening sequence** | **6017..6033** | **17** |  | Used for cloning | pHELLSGATE |
| **NOS promoter** | **6034..6217** | **184** | **=>** | NOS promoter | *Agrobacterium tumefaciens* |
| **Neomycin phosphotransferase II (NPTII)** | **6218..7039** | **822** | **=>** | Kanamycin resistance gene | *Escherichia coli* |
| **Intervening sequence** | **7040..7663** | **624** |  |  | Cloning vector pHELLSGATE |
| **NOS terminator** | **7664..7916** | **253** | **=>** | NOS terminator | *Agrobacterium tumefaciens* |
| **Intervening sequence** | **7917..7978** | **62** |  | Used for cloning | pHELLSGATE |
| **LB T-DNA repeat** | **7979..8003** | **25** | **==** | T-DNA left border | *Agrobacterium tumefaciens* |
| **Intervening sequence** | **8004-8557** | **554** |  |  | pHELLSGATE |
| **oriT** | **8558..8667** | **110** | **==** |  | pHELLSGATE |
| **Intervening sequence** | **8668..8726** | **59** |  |  | pHELLSGATE |
| **IS1** | **8727..9494** | **768** | **==** |  | pHELLSGATE |
| **Intervening sequence** | **9495..10,118** | **624** |  |  | pHELLSGATE |
| **trfA** | **10,119..11,267** | **1149** | **=>** |  | pHELLSGATE |
| **Intervening sequence** | **11,268..12,626** | **1359** |  |  | pHELLSGATE |
| **ori** | **12,627..13,215** | **589** | **<=** |  | pHELLSGATE |
| **Intervening sequence** | **13,216..14,311** | **1095** |  |  | pHELLSGATE |
| **SmR** | **14,312..15,100** | **789** | **=>** |  | pHELLSGATE |
| **Intervening sequence** | **15,101..15,774** | **673** |  |  | pHELLSGATE |

**Supplemental Table S2. Primer and PCR Information for Flanking Sequence Analysis**

| Code | Name | Sequence | Annealing Temp | PCR Product Length |
| --- | --- | --- | --- | --- |

| **1.A** | **K_Chr1_L_chr int_F** | CAGAAACTCCACAATTACCCTCAGC | **58C** | **1720bp** |
| --- | --- | --- | --- | --- |
| **1.B** | **K_Chr1_L_T-DNA_R** | GCTGTGCTCGACGTTGTCAC |  |  |
| **C** | **K_Chr1_L_T-DNA_R (SEQ)** | CAGGACATAGCGTTGGCTACC | **Sequencing only** | |
| **2.D** | **K_Chr1_L_chr end_F** | ACTAAATGAGCAAATCATAGTTCA | **58C** | **730bp** |
| **2.E** | **K_Chr1_L_chr end_R** | GTGCGTGAAAGAAGGACAACTTGG |  |  |
| **3.F** | **K_R_T-DNA_F** | CTTGTGTGCAACTCCGGGAAC | **58C** | **984bp** |
| **3.G** | **K_Chr1_R_Int chr_R** | GTGGAATGTGGAGAAGACTTGTTGGAG |  |  |
| **4.H** | **K_Chr1_R_chr end_F** | CCCATTTCGCTTGCCTCTCTTC | **58C** | **1021bp** |
| **4.I** | **K_Chr1_R_Int chr_R** | CTAGTGGTATTAAAGGTGCAATACC |  |  |
| **J** | **K_Chr3_L_chr int R** | CATGTTCAACGTGTAATATGTCAAG | **Sequencing only** | |
| **5.K** | **K_Chr3_L_T-DNA_R** | GCTGTGCTCGACGTTGTCAC | **58C** | **2131bp** |
| **5.L** | **K_Chr3_L_chr end F** | CAAACATGCTTTCTCACTGACACC |  |  |
| **M1** | **K_Chr3_L_TDNA_R** | GACGAGTTCTTCTGAGCGGGA | **Sequencing only** | |
| **M2** | **K_Chr3_L_chr int F** | GTTGATGGATCAGATTATTTACAATTC | **Sequencing only** | |
| **6.N** | **K_R_T-DNA_F** | cttgtgtgcaactccgggaac | **58C** | **1909bp** |
| **6.P** | **K_Chr3_R_chr end R** | CTTGTACTCATCCTCATCCAACAC |  |  |

**Supplementary Table S3.** **Summary of Backbone Analysis Primers and Probes.** PCR primers used for the PCR large backbone analysis and as DNA probes for small backbone Southern analysis. The location of primers within the backbone of pINVBP1 detail how the entire backbone was covered.

| **Primer name** | **Location**  **in pINVBP1** | **Sequence** | **Size of probe** |
| --- | --- | --- | --- |
| pINVBP1_BB1_FWD | 8092..8111 | GGAGAGCCGTTGTAAGGCGG | 1710bp |
| pINVBP1_BB1_REV | 9801..**9781** | AGCTCGTCCTGCTTCTCTTCG |  |
| pINVBP1_BB2_FWD | **9781**..9801 | CGAAGAGAAGCAGGACGAGCT | 1630bp |
| pINVBP1_BB2_REV | 11410..**11388** | GTTTATCGGCAGTTCGTAGAGCG |  |
| pINVBP1_BB3_FWD | **11388**..11410 | CGCTCTACGAACTGCCGATAAAC | 1573bp |
| pINVBP1_BB3_REV | 12960..**12941** | GTCGTGTCTTACCGGGTTGG |  |
| pINVBP1_BB4_FWD | **12941**..12960 | CCAACCCGGTAAGACACGAC | 1405bp |
| pINVBP1_BB4_REV | 14345..**14323** | GAGTCGATACTTCGGCGATCACC |  |
| pINVBP1_BB5_FWD | **14323**..14345 | GGTGATCGCCGAAGTATCGACTC | 1360bp |
| pINVBP1_BB5_REV | 15682..**15660** | GAGAGCAGGCTAGTTGCTTAGAT |  |
| pINVBP1_BB6_FWD | **15660**..15682 | ATCTAAGCAACTAGCCTGCTCTC | 1156bp |
| pINVBP1_BB6_REV | 1041..1022 | CATGCCAACCACAGGGTTCC |  |

**Supplementary Table S4. Primer and PCR Information Stability of Inserts Study**

| **Code** | **Name** | **Sequence** | **Annealing Temp** | **PCR Product Length** |
| --- | --- | --- | --- | --- |
| **1.A** | **K_Chr1_L_chr int_F** | CAGAAACTCCACAATTACCCTCAGC | **58**°**C** | **1720bp** |
| **1.B** | **K_Chr1_L_T-DNA_R** | GCTGTGCTCGACGTTGTCAC |  |  |
| **3.F** | **K_R_T-DNA_F** | CTTGTGTGCAACTCCGGGAAC | **58**°**C** | **984bp** |
| **3.G** | **K_Chr1_R_Int chr_R** | GTGGAATGTGGAGAAGACTTGTTGGAG |  |  |
| **5.L** | **K_Chr3_L_chr end_F** | CAAACATGCTTTCTCACTGACACC | **58**°**C** | **1712bp** |
| **M1** | **K_Chr3_L_TDNA_R** | GACGAGTTCTTCTGAGCGGGA |  |  |
| **6.N** | **K_R_T-DNA_F** | cttgtgtgcaactccgggaac | **58**°**C** | **1909bp** |
| **6.P** | **K_Chr3_R_chr end_R** | CTTGTACTCATCCTCATCCAACAC |  |  |

**Supplementary Table S5. Probe Information and Sequences Used for Expression Analysis**

| **GENE** | **nCounter Pool**  **Probe Type** | **Probe code name** | **Sequence** |
| --- | --- | --- | --- |
| NPTII | Probe A | DQ449904.1:328_T005 | CGGATCAAGCGTATGCAGCCGCCGCATTGCATCAGCAAAGACGCCTATCTTCCAGTTTGATCGGGAAACT |
| VInv vacuolar invertase | Probe A | NM_001288064.1:1547_T007 | GACCATCAGCTCCTTTAGAAATGTAGAAGTAAACTGGCGTTAGCTCAGATCCAATTTGGTTTTACTCCCCTCGATTATGCGGAGT |
| Cox1-B | Probe A | X83206.1:843_T008 | GAAGAACCAAAAGAGATGCTGGTATAATATGGGGTCTCCCCCTCCAGCGGCTTTCGGGTTATATCTATCATTTACTTGACACCCT |
| Actin | Probe A | X55749.1:2563_T009 | GACATAGCGTAATCTTAATGCATCGGACTGCTCAAAACAACTACCTTTGTCAACAGCCACTTTTTTTCCAAATTTTGCAAGAGCC |
| Elongation factor 1-alpha | Probe A | XM_006343390.2:702_T010 | GAGGGTTGGGCCCTTGTACCAGTCAAGGTTGGTAGACCTCTCAATCATGCACCGTGTGGACGGCAACTCAGAGATAACGCATAT |
| sucrose-phosphate synthase 2 | Probe A | XM_006360882.2:2555_T011 | TGTTGAGATGGCAAATCCTGATACTCTTGCGACCTGTGGATCGGACTTAACCTGGAGTTTATGTATTGCCAACGAGTTTGTCTTT |
|  |  |  |  |
| NPTII | Probe B | DQ449904.1:328_ProbeB | CGAAAGCCATGACCTCCGATCACTCCGATGTTTCGCTTGGTGGTCGAATGGGCAGGTAGC |
| VInv vacuolar invertase | Probe B | NM_001288064.1:1547_ProbeB | CGAAAGCCATGACCTCCGATCACTCCGGAGCCACTGAGGATCTAGTTTGATCAGCACAGAAGTGAGTCTCAGCTC |
| Cox1-B | Probe B | X83206.1:843_ProbeB | CGAAAGCCATGACCTCCGATCACTCCTTATGATACCGGATCCAGGCAGAATGGGAATATACACCTCTGGATGACC |
| Actin | Probe B | X55749.1:2563_ProbeB | CGAAAGCCATGACCTCCGATCACTCGAAGTGTATATATGTTCAAGATATTAGGTGGTGTGGTCCCCAAATCCGAC |
| Elongation factor 1-alpha | Probe B | XM_006343390.2:702_ProbeB | CGAAAGCCATGACCTCCGATCACTCGGTTTGTCTGTTGGCCTCTTGGGCTCATTAATCTGGTCGAGAGCATCAAG |
| sucrose-phosphate synthase 2 | Probe B | XM_006360882.2:2555_ProbeB | CGAAAGCCATGACCTCCGATCACTCTTGATGTTCCCGGATTTTAGAAATGCTGCCAATTCGGACATCGACAT TGC |

**Supplementary Table S6.**  **Sanger Sequencing Summary of Junctions**. Kal91.3 has two T-DNA inserts. The table shows the 1kb chromosomal flanking region sequence and the 500bp flanking T-DNA region sequence for each left and right border for each insert.

| **Kal91.3 Chromosomal Insert** | **Junction sequence adjacent to left border region: 1kb flanking and 500bp of T-DNA** | **Junction sequence adjacent to right border region: 1kb flanking and 500bp of T-DNA** |
| --- | --- | --- |
| Chromosome 1 Insert | **1kb Flanking** CAAAAAGGAAAAAAAATATTATTATTTTTTAAAATGAAAATTGAAGTTATGTTTGGATATGAACAATAAATTGAGTTGTTTTTGAATTTTTGAGAGTGATTTGGAGTGAAAATTTGGAAAAACTTTTTATTGAAAATTGGGTTTTAAAATTTCAAAAATATTCAAAATTCAACTTCAAGTTCCCTTTTATGGGGCCCCCACAGAATTTCGAGAGTCACTTGAATTTTAACAATTTTTTTAATTTTTTAATTTTTTAATTGTTGGGATTTATAATATTTTTAGGTAATTTTCAAATATATTCTAACTAAAAAGGAAAAGTAAGACAAACAAATTAAAACAAAAAAAAAAACACAGAAACTCCACAATTACCCTCAGCTAGAAGCAAACACGTCGACGAAGGAATGCCTAGCTACTTCTAGCCACTTCTTTAAATGATCAAATTATCCTTATCGAAGAAGCAGACATGTTGACAAAGACCTGCTAATAGTAAAATAATTCAATTTTTGTATCTATATGAAAATATTCCATTAATAATCCAAGGTACAGTCTTTCCACGTCGTTTCTAAGCACATCGATCCCTCTCATATGTTTCCAAGTTGTCCTTCTTTCACGCACAATTGACTTGTCAGAATGGAAATTTTGTTCGTAATTTGACTAGTTATTACTTCCTATATCTTCATTTAATATGTATTAATTTTTTTAAAACTTACATAAATTTCACTAATTTAGGAATTAATTATTCAGATACATTCTAATTCTAATATGTAATATTGCGAATCCTATCAGATTTTAGTGCATCCAGATATATTTAGATATGTTCAAATACATGTTTTTCGAGATACATGCAATCAAAATTAAGCGTAATTTATTCTAGATACATCGTATCCAATTGAATTTGCATGTATCGATCTAGGATACATAGACAAATCCCTCCCGCCTCCCTTCCATCTCGCTCGTCACTCTCCTATGTATCGGGTACCCCAGATTCATGTGGATCACA TDNA  **500kb T-DNA**  TDNACCAGTTGGATTAATCTTGCCTTTCCCCGCATGAATAATATGATGAATGCATGCGTGAGGGGTATTTCGATTTTGGCAATAGCTGCAATTGCCGCGACATCCTCCAACGAGCATAATTCTTCAGAAAAATAGCGATGTTCCATGTTGTCAGGGCATGCATGATGCACGTTATGAGGTGACGGTGCTAGGCAGTATTCCCTCAAAGTTTCATAGTCAGTATCATATTCATCATTGCATTCCTGCAAGAGAGAATTGAGACGCAATCCACACGCTGCGGCAACCTTCCGGCGTTCGTGGTCTATTTGCTCTTGGACGTTGCAAACGTAAGTGTTGGATCGGGGTGGGCGAAGAACTCCAGCATGAGATCCCCGCGCTGGAGGATCATCCAGCCGGCGTCCCGGAAAACGATTCCGAAGCCCAACCTTTCATAGAAGGCGGCGGTGGAATCGAAATCTCGTGATGGCAGGTTGGGCGTCGCTTGGTCGGTCATTTCGAACCCCA | **1kb Flanking** TDNAACCTAGTATGGTTCGCATGTATCTGGGATAAATTATTATCTCACTCACCTCCCTCCCCATTTCGCTTGCCTCTCTTCCTATTTCAATGTATCTAGTAACAAAAATACATGTATCTAAATTCTAGGTATATCTTCCTCAATTCTAGATAATTTTATTAATTTTAAAATAATTATAATATCAACTAATTAATAATCACTTGTTTGAATGCTTCATTATTGGTTGTTTGACTCGAAGACTTGTTGATTTTAAAGAGTTTTTTTTCTTCTTCCTCTTAAATTGTATGTATTAAGTCAAGAAGACTCCAACAAGTCTTCTCCACATTCCACAACACTATTAAGGTCCCATTTGAATTTGATTTGAAATCAGATTGATTTGAAGTTAAAGTTAAGATTTGGGATATGTAAATAAAATATAAGAACGTATCCTATCAGTCATTCCCAAAAAAATTGCCTTTATTCGCTGCAAAAGGGCAAAGAAATGTTGATTTTAAAAAATGAAATGGAAAAATTAAGAATTACAAAATAAGGGTTTAAAAGAGTTAAAAAGAAAAGTATATACTTATATATAATAGAATAATATCTTCTTTCACGAAAAGATTATGAAAATTGAAGTTATGTTTGAAATGACTTTCTGTTACAACACCTTCCAACTTATTGTTAGTATTGAATTTCCAGTAAGTTTATTACCATCTAGTCTCAGTTCCATTAATGAAGACGTATCTCCCAATCCCATTGAAAAGAAGTTACTCCTACCTTTGAATAATTCAGATACTTGTCCCTCAAATTGATTTTTTGAAAGAGAAAGAAATTCTAATTTCCCTAAAATTCCAATTACATTTGGTAACTTTCCAGTAAGCATATTACCAGATATGTCAATATTAGTTATTGAGCTCAAATTTGAAATCAGACTTGAAACTGCTCCCTCAAGACGGCTTTCACTAAGGTAAAGTGATTCTAGATCTTTGCATCGATAGAGCCAATTTGGTATGGTGGAGTTAAGG  **500kb T-DNA**  CAGATTTTTGTGGGATTGGAATTAATTCGTCGAGCGGCCGCCTGCAGGTCGATATGGGAGAGCTCCCAACGCGTTGGATGCATAGCTTGAGTATTCTATAGTGTCACCTAAATAGCTTGGCGTAATCATGGTCATAGCTGTTTCCTGTGTGAAATTGTTATCCGCTCACAATTCCACACAACATACGAGCCGGAAGCATAAAGTGTAAAGCCTGGGGTGCCTAATGAGTGAGCTAACTCACATTAATTGCGTTGCGCTCACTGCCCGCTTTCCAGTCGGGAAACCTGTCGTGCCAGCTGCATTAATGAATCGGCCAACGCGCGGGGAGAGGCGGTTTGCGTATTGGGGCTGAGTGGCTCCTTCAACGTTGCGGTTCTGTCAGTTCCAAACGTAAAACGGCTTGTCCCGCGTCATCGGCGGGGGTCATAACGTGACTCCCTTAATTCTCCGCTCATGATCAGATTGTCGTTTCCCGCCTTCAGTTTAAACTATCAGTGTTT TDNA |
| Chromosome 3 Insert | **1kb Flanking**  CAATTTTCACATATTCAAAACATCCAAAAAACGAATTGATATAATGTGAATCTAAATTCAAAAAACTTAAAAAGAATAAAAACTAAATTAATTTGATTTCTATTTTCACTTTTATCAAATCAAGAATTTAAGAATTAAATCAAACCAACTAAATATTCCCTCCTAATCATGCTACTTCCCACAGGGTTATATTTTGTCTTTTGCATTACCAACAAAGATCACAAGGAAGTTGATCATTATCGAAAAATTATTTGATGAGAAATCTCCAATAATATATTATCATTATCCTATAGTATAAAAAGTTATTGTAGTTTTCGTCAAAAAATGGGGAGTCATTGAGTTTGAATTATTATAATCTTTTTAATATCACCTCATATTTTTTGATTTAAATTTATTTATTTGTTTTTTGACTTGACACCATTTTTTTTTTAAAAAAAGGCTTTTAAATTTTATGGCCCTAAAATAAGTAGAATATACTAAAATACTTTTAGTTTTTTTAAATTTAAAATGCTATGTGAAAAAATTAAAATTATAAAACTATAAAAAAAAACATTATTATTAGAATTAACTAAACTAAAAATGAAAATAAAAATAAAACAGATACATACATTAAAACTAATTAAGTAAAATTGTTAAATTAGGTCTTAGGCCTAACTCACACCCCAAAAGCTAGCTCAAAGAGAAGAGGATTGTCTAAGCCTTATAAGGAGTCCACTCATTTCATTAACCACCGATGTGGAACTTTTGTCATTCTTTAACAAAAATATTGATAATATAGTAAAAGTTCCAATCGATCCATTTGTAGCAAAAGAAGTGTGCTAAAAAGGCATTAGGAGGCAAATAGTTTTTTTGAAAAGAAAATAAAAGCATAGTAATAAATAAATGTTCTTTTTAATTTCACTTCAACTAACAGAGCTTTATACTCACTTAAATTAAAATAAATAGACATATATATCTTACTTGACATATTACACGTTGAACACGTAAATAAATTATCACA TDNA  **500kb T-DNA**  TDNACAAATGTTTGAACGATCTGCTTCGACGCACTCCTTCTTTACTCCACCATCTCGTCCTTATTGAAAACGTGGGTAGCACCAAAACGAATCAAGTCGCTGGAACTGAAGTTACCAATCACGCTGGATGATTTGCCAGTTGGATTAATCTTGCCTTTCCCCGCATGAATAATATTGATGAATGCATGCGTGAGGGGTATTTCGATTTTGGCAATAGCTGCAATTGCCGCGACATCCTCCAACGAGCATAATTCTTCAGAAAAATAGCGATGTTCCATGTTGTCAGGGCATGCATGATGCACGTTATGAGGTGACGGTGCTAGGCAGTATTCCCTCAAAGTTTCATAGTCAGTATCATATTCATCATTGCATTCCTGCAAGAGAGAATTGAGACGCAATCCACACGCTGCGGCAACCTTCCGGCGTTCGTGGTCTATTTGCTCTTGGACGTTGCAAACGTAAGTGTTGGATCGGGGTGGGCGAAGAACTCCAGCATGAGATCCC | **1kb Flanking**  TDNAACCCCTATACCCCAATTCCTCATCTCTCTTTTTCTGTGTCAGCCTTTTCTCTGTCCATATACGCCACATGGTCCATTTAGCCCCTCACCCTCCGCATCAGACAACGAACCAACACGGGTTCGGTTCGGGTGTATTTACTTTTTCCGGATCCAAGTATGTACACAGTAGTTTCCTGTATGTTTACGACTATTCTCAAACTTTTCAGTCCACTACGTTTCTTCTTCTCGCTTTTGCCTAATTTGCCTTTCTCTCTCTCTCCATCTGACTTTCCTAAAAACAATGGCAACGATCATATCTTCCATTCTCATCTAATCCATTCTTCAACTCTCTTTTGGATTTTGAATTTTTTTCCTTCTTCTTCAACTTACAAAACCCTAAACTCATTTTCACCTCGATCTCACTTCTTCTTGCTGTTTTCTAGACTGTTTTATTGGTTGAGCTATTGTAGTTTGAAGAAATCCAGCTGTATTTTGCATTCTGGTGGTGATTATTCTACTACATTAAGTGAATTATTGATGTATTTGTAGAAGAGAGTTAATTTGAGATCAATTGAACTGTTGAATTGGGAATTTTGTAGAGAGATCTGTTTTACGGGTGAATTTGGTGTACTTTGAAGTGAAGGGTGGAATTGGAGGGTTTTGGATCCGGATCAGGGAGAAACAGCGCCATTAGATGGCGCTGTTCAGACGGTTCTTCTATAGGAAGCCGCCGGATCGGCTTTAGAGATCTCTGAGCGGGTTTATGGTAAACGCTGTTTTTCTAGCCTGGAAGTTTGTAATTTTTGTGTTTTATTCTGTTGTTGAGCTTATGAACTGTGTTAAGAATAGTTTCCAGTAGCTTCTTGTTTCTGCATTTTACTTACTTTTTATCATGTTTCCTTGTTGATCGATATCCCTATTAGCTACATTTGGAATATCAGCCGCTGCTTTTCGATGTAAGTGAAGTCCTAGACTAGAGGAAATGCAACTCCTTGCGATAAAATGTGCTAGTACCCTCATTT  **500kb T-DNA**  TCAGATTTTTGTGGGATTGGAATTAATTCGTCGAGCGGCCGCCTGCAGGTCGATATGGGAGAGCTCCCAACGCGTTGGATGCATAGCTTGAGTATTCTATAGTGTCACCTAAATAGCTTGGCGTAATCATGGTCATAGCTGTTTCCTGTGTGAAATTGTTATCCGCTCACAATTCCACACAACATACGAGCCGGAAGCATAAAGTGTAAAGCCTGGGGTGCCTAATGAGTGAGCTAACTCACATTAATTGCGTTGCGCTCACTGCCCGCTTTCCAGTCGGGAAACCTGTCGTGCCAGCTGCATTAATGAATCGGCCAACGCGCGGGGAGAGGCGGTTTGCGTATTGGGGCTGAGTGGCTCCTTCAACGTTGCGGTTCTGTCAGTTCCAAACGTAAAACGGCTTGTCCCGCGTCATCGGCGGGGGTCATAACGTGACTCCCTTAATTCTCCGCTCATGATCAGATTGTCGTTTCCCGCCTTCAGTTTAAACTATCAGTGTT TDNA |

**Supplementary Fig. S1 Eurofins Technical Sheet**
